# From functional food to medicinal product: Systematic approach in analysis of polyphenolics from propolis and wine

**DOI:** 10.1186/1475-2891-8-33

**Published:** 2009-07-22

**Authors:** Marica Medić-Šarić, Vesna Rastija, Mirza Bojić, Željan Maleš

**Affiliations:** 1 Department of Medicinal Chemistry, Faculty of Pharmacy and Biochemistry, University of Zagreb, A. Kovačića 1, 10000 Zagreb, Croatia; 2 Faculty of Agriculture, Josip Juraj Strossmayer University of Osijek, Trg Sv. Trojstva 3, 31000 Osijek, Croatia; 3 Department of Pharmaceutical Botany, Faculty of Pharmacy and Biochemistry, University of Zagreb, Schrottova 39, 10000 Zagreb, Croatia

## Abstract

In the last decade we have been working on standardization of propolis extract and determination of active constituents of wine those are rich in polyphenolics and have nutritional as well as therapeutic value. Here we are summarizing our results and providing overview on systematic approach how to analyse natural products rich in flavonoids and phenolic acids.

Chromatographic methods (thin layer chromatography and high performance liquid chromatography) were used for identification, quantification, and characterization of individual flavonoid or phenolic acid. Total content of active constituents and antioxidant activity were determined by spectrophotometry. Pharmacokinetic parameters were determined by high performance liquid chromatography and using appropriate software. Quantitative structure-activity relationship study of antioxidant activity was conducted, as well as assessment of prolonged propolis supplementation on antioxidative status of organism.

Thin layer chromatography-densitometry has been proven as quick and reliable method for standard analysis of propolis and wine; the best mobile phase being chloroform – methanol – formic acid (98–100%) in ratio 44 : 3.5 : 2.5 (*v/v*). Higher number of polyphenolics was determined by high performance liquid chromatography; 15 compared to 9 by thin layer chromatography. Interactions *in situ *with acetylsalicylic acid were detected with most of polyphenolics analysed. Plasma protein binding and blood-barrier penetration was greatest for flavone. The interactions with human serum albumin have been grater than 95% for all flavonoids analysed. The prolonged propolis consumption increased superoxide dismutase activity.

The necessity of standardization of natural products and their registration as functional nutraceuticals demand easy, quick and inexpensive methods of analysis. In this work we provided overview of analytical part for polyphenolics that could be used as data for possible registration of final products either as functional food or medicinal product.

This feature introduces the readers to the authors' research through a concise overview of the selected topic. Reference to important work from others in the field is included.

## Background

The main causes of death in western countries are cardiovascular diseases and cancer. Over 50 percent of the population has some kind of chronic condition (high blood pressure, high cholesterol, arthritis, diabetes, asthma, osteoporosis...), so the goal of the many researches in the last decades is improving the quality of life. Much of the interested was transfered to homeopathy, alternative, and folk medicine, evincing polyphenols as one of the main nutraceuticals [1].

The most abundant sources of polyphenols, mainly flavonoids and phenolic acids, are propolis and wine. Flavonoids and phenolic acids have antibacterial, antifungal, antiviral, antineoplastic, hepatoprotective, immunomodulating, and antiinflammatory properties. Their use has been proven beneficial in allergies, asthma, diabetes, hypertension, micro bleeding, etc. Much of these pharmacological effects can be associated with antioxidant activity. Hence, antioxidant activity is the most studied property of polyphenols [2].

In the last decade, we were working on several projects with the aim of developing standardized extracts of propolis and determination of active constituents of wine, transferring knowledge from the laboratory to everyday-practice for the purpose of analyzing natural products. To accomplish this we used rather simple, reliable, and quick spectrophotometric and liquid chromatography methods for analysis of polyphenols, the results of which are reviewed here. These were used to suggest mixtures of propolis that will provide the best final product.

In this review we wanted to systematize the approach in analysis of polyphenols rich samples providing mostly unpublished data (Published data is marked as reference). Some basic concepts of optimization of chromatographic system, chromatographic parameters describing pharmacokinetic and quantitative structure-activity relationship are described. The use of liquid chromatography for different objectives is thoroughly illustrated: identification, quantification, characterization; ADME study (Absorption, Distribution, Metabolism, Excretion) and interactions. To demonstrate *in vivo *antioxidant effects of polyphenols from propolis preliminary clinical trial of prolongated propolis is presented.

### Flavonoids and Phenolic Acids. Propolis and Wine

**Figure 1:**
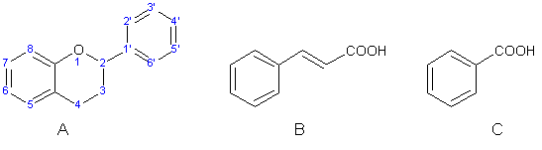
**Basic structures of flavonoids (A) and phenolic acids (B-cinnamic acid, C-benzoic acid)**.

Flavonoids and phenolic acids are plant polyphenols characterized by having one or more phenolic hydroxyl groups or carboxylic group in phenolic acids. The major classes of polyphenols are phenolic acids (hydroxycinnamic and hydroxybenzoic acids), flavonoids, anthocyanins, and tannins (Figure 1) [2,3].

**Figure 2:**
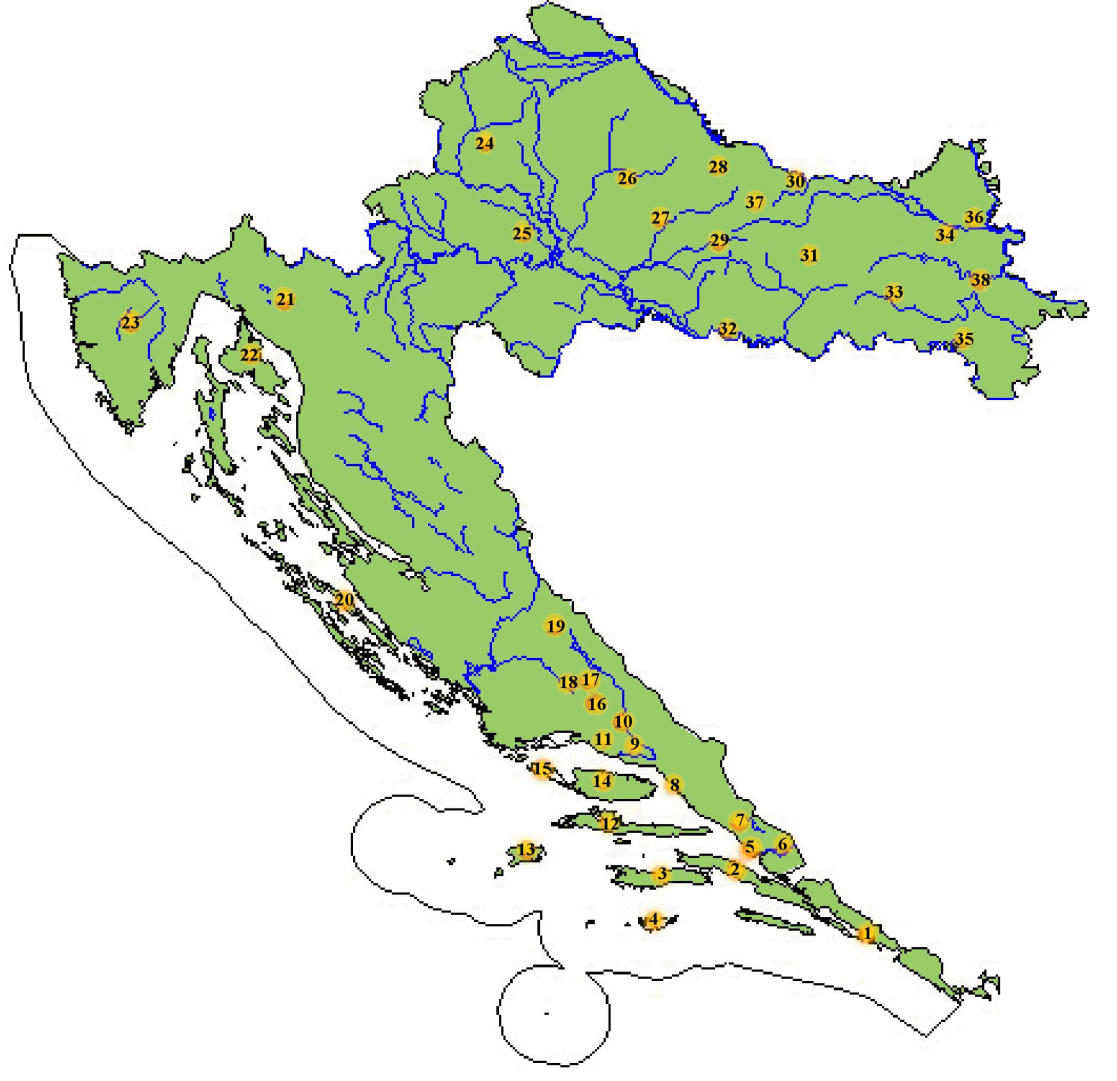
**The map of Croatia**. Areas of origin of propolis samples (marked by numbers).

Propolis is a resinous substance collected by honeybees (*Apis mellifera*, L.) from a variety of plant sources. Its chemical composition is very complex and varies with geographic origin, depending on the local flora and phenology of the source plants. The benefits of effects of propolis on human health were recognized thousand of years ago. It has wide range of biological activities including antibacterial, antiviral, antiinflammatory, and antioxidative [4]. To illustrate the regions where our propolis samples originate, the map of Croatia is provided on Figure 2.

Wine is an alcoholic beverage that contains a large amount of different polyphenols extracted from grapes during the processes of vinification. These molecules are responsible for color, acerbity, flavor, and antioxidant properties of wine [5].

### Optimization of Chromatographic Systems [6]

The purpose of optimization of chromatographic systems is to find the one that shows the greatest difference in identification characteristics between substances e.g. retention factor (*R*_F_) value in thin layer chromatography.

To accomplish this numerical taxonomy methods were used. Taxonomic entities are polyphenols and their numerical characteristic is *R*_F _value. Matrix of resemblance of *R*_F _values is analyzed to find the mobile phase in which the resemblance is the lowest and separation of analyzed substances is optimal. In short, the matrix of *N *substances and *t *mobile phases is reduced using different algorithms to form a cluster of similar mobile phases, the procedure is repeated until the final cluster including all mobile phases is formed and the results are presented in form of dendrogram.

Complementary mathematical methods are used. If the *R*_F _values are distributed into groups of width *E*, in *R*_F _units, then in group k *n*_k_*R*_F _values are present, and the average information content is described by Shannon equation:

(1)

with the assumption that substances with the *R*_F _value in the same group can not be identified. The higher the average information content is, the better results of separation for mobile phase are. To positively identify two substances difference between *R*_F _values has to be greater than *E *(usually 0.05). Discriminating power (*DP*) is used as a measure of the effectiveness of chromatographic systems. The *DP *of a chromatographic system is the probability of separating two flavonoids (or phenolic acids) selected at random from a specific substance population. Two flavonoids (or phenolic acids) are chromatographically similar if the differences in their identification values do not exceed the error factor *E*. In case of mixture of eluents *DP *is defined as probability that two randomly selected substances can be distinguished by one mobile phase. Mathematically it can be expressed by the following equation:

(2)

where *k *is the number of mobile phases, *N *number of substances analyzed and *M *the total number of matching pairs. The average number of similar substances *T*, for each mobile phase, is defined by expression:

(3)

The highest *DP *and the smallest *T *value are attributed to the most appropriate combination of eluents.

This procedure of optimization has been used in the analysis of numerous plant samples: *Zizyphus jujuba *Mill., *Chamomilla recutita *(L.) Rauschert, *Rosmarini folium*, *Guiera senegalensis *J.F.GMEL., *Rhamni cathartici fructus*, *Lavandulae flos*, *Sambuci flos*, *Helleborus atrorubens *Waldst. et Kit. etc. [7-14].

### Chromatographic Parameters Describing Pharmacokinetics of Polyphenols [15]

As our samples are consumed orally absorption and distribution of polyphenols are of interest. Absorption through the gastrointestinal tract (GIT) membrane, plasma protein binding and the penetration through blood brain barrier (BBB) was studied using *in silico *approaches and liquid chromatography.

In reverse phase thin layer chromatography (RP-TLC), the chromatographic parameter *R*_M _is used to describe the lipophilicity of a substance:

(4)

It represents a logarithm of ratio of spot distance from the front and from the start. The higher the value of *R*_F_, chromatographic parameter is lower and the substance is more polar. As *R*_M _depends on the percentage of the organic modifier in mobile phase, *R*_Mw _value with 0% of organic modifier is used and can be extrapolated using the equation:

(5)

where *b *is the coefficient of the slope and *Φ *is the concentration of the organic modifier.

In reverse phase high performance liquid chromatography (RP-HPLC) log *k *is used as a measurement of distribution between hydrophobic (stationary) and hydrophilic (mobile) phase:

(6)

The values of log k are compared at a 0% concentration of organic modifier based on the equation:

(7)

**Figure 3:**
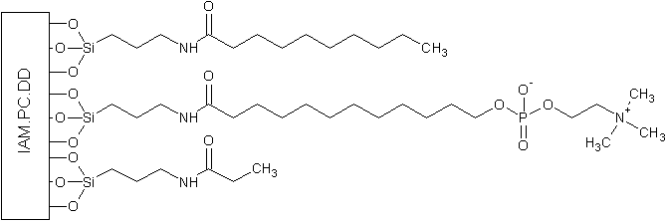
**The structure of stationary phase of immobilized artificial membrane – phosphatidylcholine – drug-discovery column (IAM.PC.DD)**.

As mucosa membranes are modified with phospholipids that are changed under physiological pH due to interaction, absorption and distribution of polyphenols can be changed, too. To predict this interaction immobilized artificial membrane (IAM) columns are used (Figure 3). The measurement of these interactions as well as for RP-HPLC is log *k*_wIAM_.

To assess the binding of polyphenols to the plasma protein columns containing human serum albumin (HSA) and alfa1-glycoprotein acid are used. The percentage of polyphenol bounded to HSA is calculated using the equation:

(8)

If the polyphenol shows a binding of over 95% it can potentially interact with other drugs of high affinity to HSA as the concentration of active (free) drug can increase significantly (e.g. 5% to 8% is increase of 60% in active form of drug).

### Quantitative Structure-Activity Relationship [16]

Quantitative Structure-Activity Relationship (QSAR) represents the analysis in which the structure of molecules is quantitatively correlated to biological activity. QSAR is now an irreplaceable technique in the rational design of drugs. The main postulate of QSAR is: biological activity is the consequence of chemical structure. To characterize the structure, different topological indices and three-dimensional (3D) descriptors are used. The most common topological indices are described in Table 1.

**Table 1:** The most commonly used topological indices.

**Index**	**Expression for calculation**	**Observation**
**Wiener index**		*D*_*i*,*j *_represents off-diagonal elements of matrix which stands for the shortest distance in term on number of bonds between atom *i *and *j*
**Valence connectivity index**		*v*_*i *_and *v*_*j *_are weights (valence delta values) of vertices *i *and *j *making up edge in vertex weighted graph *G*
**Balaban index**		the average distance sum connectivity; where *E *is the number of edges, μ is cyclomatic number of *G *and *ds*_*i *_is a distance sum
**Information-theoretic index**		modified Shannon's equation where *n *is the number of different sets of elements, *N*_*i *_is the number of elements in the *i*-th set of elements and the sum is over all sets of elements
**Shultz index**		molecular topological index (MTI) is based on adjacency matrix (*A*), the distance matrix (*D*) and the valency matrix (*v*); the of elements *e*_*i *_of the row matrix *v *[*A*+*D*] gives Shultz index

Besides QSAR, evolution in rational design has been achieved by using software for assessing physicochemical properties and parameters of bioavailability.

## Review

### Thin Layer Chromatography

**Figure 4:**
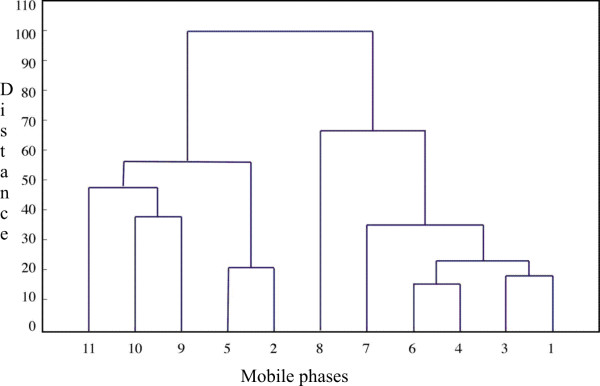
**Dendrogram for eleven TLC mobile phases used fot determination of polyphenols in samples of Croatian propolis**.

Thin layer chromatography of standard solutions of flavonoids and phenolic acids was performed on silicagel plates 60 F_254 _using different mobile phases (Table 2) and was visualized under short and long ultraviolet (UV) light after spraying with 1% AlCl_3_. The optimization was done using program KT1 [17]. Eleven mobile phases, for separation of flavonoids and phenolic acids were analyzed. The most suitable mobile phases were number 7 (*n*-hexane : ethyl acetate : acetic acid (glac.) = 31 : 14 : 5 *v/v*) and number 11 (chloroform : methanol : formic acid = 44 : 3.5 : 2.5 *v/v*), as shown by dendrogram (Figure 4) and confirmed by values of discriminating power and the average information content (Table 3) [17]. Using mobile phases 11 and 7 we succeeded to identify up to 9 polyphenols in samples of Croatian propolis.

**Table 2:** The mobile phases studied.

**No**	**Solvents**	**Proportions (*v/v*)**
**1**	toluene – ethyl acetate – formic acid (98–100%)	36 : 12 : 5
**2**	cyclohexane – ethyl acetate – formic acid (98–100%)	30 : 15 : 5
**3**	toluene – ethyl acetate – glacial acetic acid	36 : 12 : 5
**4**	cyclohexane – ethyl acetate – glacial acetic acid	31 : 14 : 5
**5**	*n*-hexane – ethyl acetate – formic acid (98–100%)	31 : 14 : 5
**6**	toluene – acetone – formic acid (98–100%)	38 : 10 : 5
**7**	*n*-hexane-ethyl acetate – glacial acetic acid	31 : 14 : 5
**8**	petroleum ether (40–70°C) – ethyl acetate – formic acid (98–100%)	30 : 15 : 5
**9**	carbon tetrachloride – acetone – formic acid (98–100%)	35 : 10 : 5
**10**	*n*-hexane – ethyl acetate – glacial acetic acid	30 : 20 : 1.5
**11**	chloroform – methanol – formic acid (98–100%)	44 : 3.5 : 2. 5

**Table 3:** *DP *and *I *output date for error factor *E *= 0.03 for each mobile phase.

**Mobile phase**	***DP***	***I *(bit)**
1	0.8538	3.221
2	0.7836	2.735
3	0.8655	3.076
4	0.8947	3.616
5	0.8187	2.860
6	0.8480	3.011
**7**	**0.9298**	**3.682**
8	0.7895	3.050
9	0.9240	3.511
10	0.9181	3.471
**11**	**0.9415**	**3.827**

**Figure 5:**
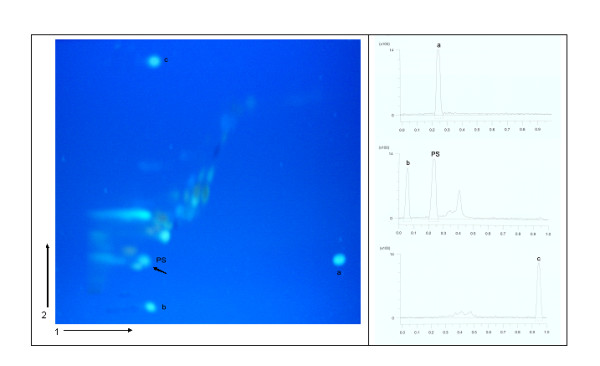
**2D-TLC of caffeic acid in propolis sample from Peruča and standards of caffeic acid**. The plate was first developed in the direction 1 then 2 using mobile phase 1 (n-hexane : ethyl acetate : glacial acetic acid = 31 : 14 : 5 *v/v*) and mobile phase 2 (chloroform : methanol : formic acid = 44 : 3.5 : 2.5 *v/v*). Bands were visualized under short and long wave UV light and under long wave UV light after spraying with 1% AlCl_3 _ethanol solution. Concetration obtained from chromatograms: a = *m*_max _= 2.50 μg, b = *m*_min _= 0.83 μg, c = *m*_mid _= 1.67 μg, PS = *m*_propolis sample _= 2.91 μg [18].

Analysis of flavonoids and phenolic acids in propolis was performed by two-dimensional thin layer chromatography (2D-TLC) using optimized chromatographic systems and detection and quantification was done using CAMAG Reprostar 3 densitometer with CAMAG VideoScan TLC/HPTLC evaluation software. The absorbance was recorded at 254 and 366 nm, the later being the wavelength of quantification [18]. Since propolis is a rather complex mixture for simple TLC, 2D-TLC analysis of propolis samples previously optimized chromatographic systems were used. Plate and densitograms of standard solution of caffeic acid and propolis sample from Peruča are presented in Figure 5.

2D-TLC was shown to be a suitable method for quantitative determination of flavonoids and phenolic acids in Croatian propolis samples. The linear relationship between absorbance and mass of standard was established:

(9)

The presence of coumaric and cinnamic acids could not be established, as these compounds do not form fluorescent complexes with AlCl_3_.

**Figure 6:**
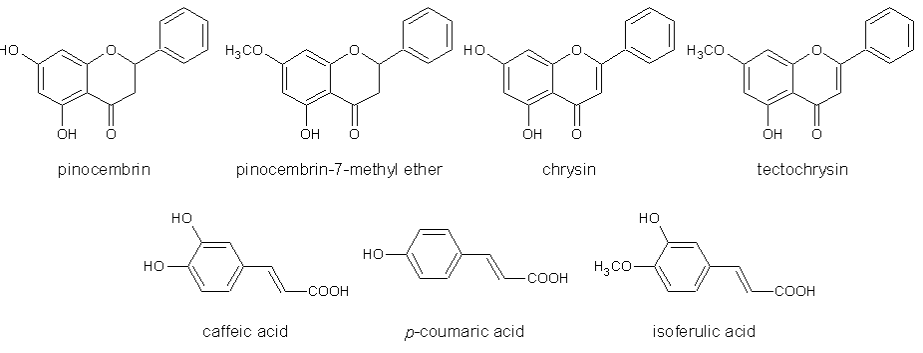
**Structures of flavonoids and phenolic acids quantified in propolis tinctures**.

For the quantification purpose high performance thin layer chromatography (HPTLC) under previously optimized conditions was used. Seven polyphenols (Figure 6) have been quantified and the method was validated for a routine control of propolis tinctures [19]. HPTLC method was used for quantification and the results of validation are shown in Table 4. HPTLC method was applied to 7 propolis samples and results are shown in Table 5.

**Table 4:** Results of validation of HPTLC method [19].

**Validation data**	**Value**
linearity	r > 0.99
precision	
repeatability of the sample application and intra-day precision	RSD < 3.8%
repeatability of peak-area measurement	RSD < 2.4%
accuracy	RSD < 6.5%
limit of detection	PME 7.5 ng/band IFA 7.5 ng/band CA 60 ng/band
limit of quantification	PME 22.5 ng/band IFA 22.5 ng/band CA 180 ng/band
robustness	
inter-day precision and stability of standard solutions	RSD < 5% (two days)
stability on the plate	RSD < 3.4% (up to 2 hours)*
effect of temperature	RSD < 4.6% (20/26°C)
selectivity	absorption spectra matching > 0.99

*only caffeic acid degraded up to 9.4% in 120 minutes, but as the analysis lasts less, this was not of great significance; CA: caffeic acid; IFA: isoferulic acid; PME: pinocembrin-7-methylether; RSD: relative standard deviation.

**Table 5:** Phenolic acid and flavonoid content (expressed as mean of mass concentration in mg/mL) of the Croatian propolis samples analyzed [19].

**propolis sample\polyphenolic**	***p*-coumaric acid**	**caffeic acid**	**chrysin**	**tectochrysin**	**pinocembrin**	**pinocembrin-7-methyl ether**	**isoferulic acid**
**Veliki Zdenci**	0.3223+1.32	0.1204+0.99	-	-	-	-	-
**Sisak**	0.1331+8.22	0.2675+0.22	0.1423+4.09	0.0845+2.90	-	-	-
**Labin**	0.1464+3.82	0.3000+0.74	0.3987+3.30	-	-	-	-
**Čisla (Omiš)**	0.2047+2.09	0.4123+0.51	0.1247+1.84	-	-	0.0593+7.36	-
**Pelješac**	-	0.4196+0.38	0.4896+1.46	0.6775+1.45	0.8454+2.13	0.7452+0.84	0.5068+0.59
**Metković**	0.2968+1.04	0.3538+0.79	0.5616+2.62	1.1816+0.81	0.6725+1.54	1.1621+0.85	0.4376+2.42
**Mixture of propolis**	0.1908+2.14	0.3398+0.92	0.6237+4.47	0.4179+1.71	1.1465+3.24	0.2305+5.49	0.4109+4.54

For the investigation of ADME for flavonoids reversed-phase thin layer chromatography was used. Chromatography was performed on RP-18F_254s _plates with binary mobile phase prepared from methanol and phosphate buffer. The pH was at least 2 units lower than p*K*_a _value of phenolics, and organic modifier was varied from 30 to 80% in 5% increments. ADME data was calculated using the following programs: CLOGP, KowWin, Molinspiration, MolSoft, XLOGP, Osiris, VEGA ZZ, DRAGON, ChemSilico, ALOGPS, Interactive Analysis, SPARC [20]. Regular retention behaviour was observed for all the polyphenols investigated – a liner decrease of retention with increased concentration of organic modifier of mobile phase, with standard deviation of less than 0.1. To compare values of retention of polyphenols *R*_Mw _values were extrapolated to 100% buffer in mobile phase by using the equation:

(10)

where *Φ *is the concentration of organic modifier. *R*_Mw _values of flavonoids were in the range 2.309–3.580 and phenolic acids 1.318–2.176. The correlation between *R*_Mw _and *Φ*_0 _(*R*_M _= 0) was established:

(11)

It is evident that *R*_Mw _and *Φ*_0 _are well correlated but are not identical. This is the case with isomers sakuranetin and isosakuranetin that have the same chromatographic lipophilicity *R*_Mw _= 3.111, but different *Φ*_0_, 19.61 and 20.22 respectively.

Most of the used programs are "blind" to this difference occurring between structural isomers, and for MLOGP even different classes of flavonoids could not be distinguished: galangin (flavonol) and apigenin (flavone).

Besides lipophilicity, that is the main determinant for ADME, using ChemSilico software plasma protein binding, blood-brain barrier penetration and human absorption were predicted (Table 6).

**Table 6:** Results obtained using ChemSilico software [20].

**ADME data**	**Results**
plasma protein binding	
- flavonoids	85.41% (myricetin) – 95.65% (flavone)
- phenolic acids	70.22% (sinapic acid) – 85.99% (cinnamic acid)
blood-brain barrier penetration	negative except flavon and flavanone
human absorption	significant

BBB penetration was positive only for flavone and flavanone, as these do not have hydroxyl groups. Human absorption was the lowest for myricetin (58%).

Surprisingly, correlation of *R*_Mw _and calculated log *P *values was rather poor, and the best correlations were found between *Φ*_0 _and XLOGP values for flavonols, and human intestinal absorption calculated by ChemSilico and chromatographic RMw (for flavanones) and *Φ*_0 _(for phenolic acids) values.

**Figure 7:**
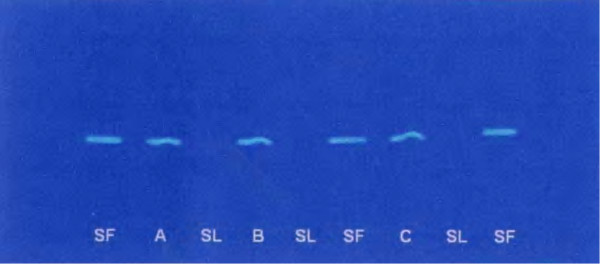
**Chromatogram of interaction of flavonoid pinocembrin and ketoprofen**. Interaction of flavonoid pinocembrin and ketoprofen obtained under long wave UV light. SF – standard of flavonoid (0.33 mg/mL), A, B, C – interaction lines of pinocembrin and ketoprofen in concentration 0.5, 1.0 and 1.5 mg/mL respectively, SL – standard of ketoprofen in the same concentration as in A, B and C.

Based on the procedure developed by Ramić *et al*. [21] chemical interactions were studied using the optimized chromatographic system for polyphenols. Interactions of 19 polyphenols and over-the-counter (OTC) analgesic (acetylsalicylic acid, paracetamol, diclofenac, ibuprofen and ketoprofen) and vitamins (C and E) were examined. Interactions were detected between acetylsalicylic acid and acacetin, galangin, chrysin, pinocembrin and cinnamic acid; ibuprofen and flavone, ketoprofen and pinocembrin, vitamin C and myricetin, morin and quercetin; and vitamin E with flavanone. Example of chromatogram obtained is shown on Figure 7.

These chemical interactions cannot be replaced *in vivo *analysis, but rather be used as basis for further research of phenolics in biological systems.

**Figure 8:**
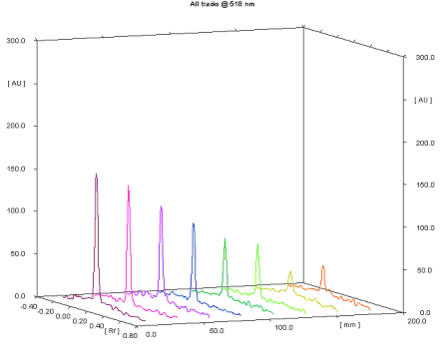
**Band-blot test of polyphenols present in propolis using the stable free radical DPPH·**. Chromatogram obtained after spraying the layer (applied polyphenol standard mixture bands in the range from 45 (track 1) to 10 μg/mL (track 8)) with 0.3 mM solution of DPPH^· ^radical. *AOE *of standard mixture is 1.89 (*H *= 1.89*c *+ 22.856, *r*^2 ^= 0.974) [22].

Band-blot test was used to evaluate antioxidant activity of propolis samples compared to standard mixture of caffeic acid, galangin and pinocembrin using stable free radicals: 2,2'-azino-bis(3-ethylbenzothiazoline-6-sulphonic acid) (ABTS^·+^) and the 2,2-diphenyl-1-picryl-hydrazyl (DPPH^·^) [22]. Band-blot test was performed instead of original dot-blot test using the stable free radical of DPPH^·^. The reason for replacing dots with bands is equal to the distribution of samples in a line instead of over-concentrated centre of spot that can not be easily quantified. Antioxidant efficacy (AOE) was expressed as the slope of the dose-response curve of decrease in DPPH^· ^radical absorbance was used. The chromatogram for standard mixture is shown on Figure 8. Band-blot test has proven a rather quick method for assessing antioxidant activity of a great number propolis samples as the only application of samples and visualization after spraying was done, taking less than 10 minutes.

**Figure 9:**
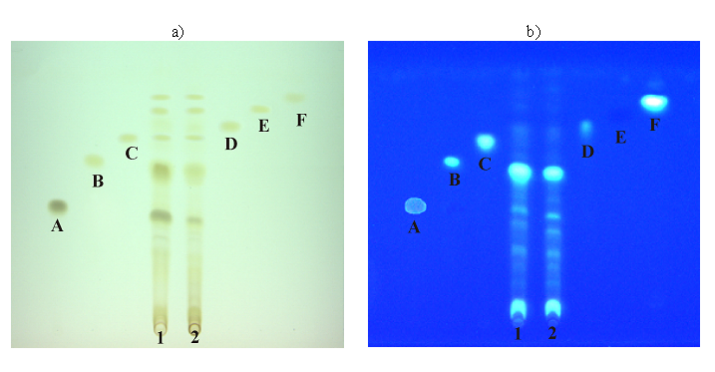
**Chromatograms of extracts of red wines "Merlot" (1) and "Frankovka" (2) by use of mobile phase benzene : ethyl acetate : formic acid = 30 + 15 + 5 *v*/*v*/*v***. a) recorded under UV light λ = 254 nm; b) recorded under UV light λ = 366 nm, after spraying with 1% ethanol solution of AlCl_3_. The standard substances used: A – gallic acid, B – caffeic acid, C – apigenin, D – kaempferol, E – *p*-coumaric acid, F – naringenin [23].

**Figure 10:**
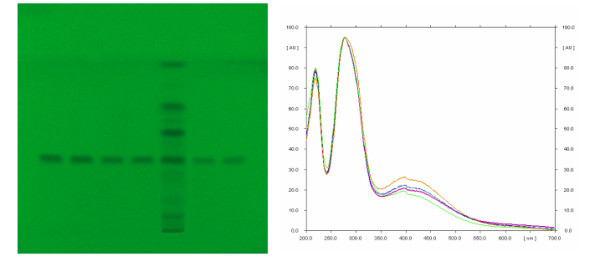
**TLC identification and quantification of gallic acid**. Photograph of the chromatographic plate recorded at UV light λ = 254 nm, for the purpose of quantification of gallic acid in the sample of wine, with the spectrum of marked peaks obtained using CAMAG densitometer.

For wine samples the same procedure of optimization was used to select the most appropriate mobile phase (benzene : ethyl acetate : formic acid = 30 : 15 : 5 *v/v*). Six phenolic compounds were indetified in the tested samples. The plates are shown on Figure 9[23]. TLC quantification of polyphenols in wine was performed using CAMAG system. The substances were identified on basis of *R*_F _values and UV spectra. Densitometric quantitative analysis of gallic acid in wine extract is presented on Figure 10, as an example.

### High Performance Liquid Chromatography

HPLC analysis of propolis was conducted on Agilent 1100 using X-bridge C18 column. Binary mobile phase of eluents A (10 mM ammonium-formiate in water) and B (10 mM ammonium-formiate in methanol) both of pH = 7 (adjusted with formic acid) was used with the gradient stated in Table 7[22]. Flow rate was 0.5 mL/min at room temperature. 21 standards of polyphenols were used; their identification was based on retention time and UV spectrum, and quantification was done at 270 nm.

**Table 7:** Gradient of mobile phases used for HPLC analysis of propolis.

time (min)									
									
mobile phase	0	15	25	30	40	50	60	75	80
%A	90	50	50	40	40	10	10	90	90
%B	10	50	50	60	60	90	90	10	10

Reverse phase HPLC was used for the lipophilicity/hydrophobicity of flavonoids and phenolic acids. Mobile phase A was 2% acetic acid in water and B 2% acetic acid in methanol. Gradient method started with 95% of mobile phase A and 5% of mobile phase B that was linearly increased to 100% during 20/60 minutes [24]. As shown on chromatogram, phenolic acids as more polar substances were first eluted from the column, followed by more hydrophilic and lipophilic flavonoids. HPLC results were used to identify flavonoids and phenolic acids present in propolis samples, to quantify the content of polyphenols, to explain the antioxidant activity *in vitro *and *in vivo *as well as they were used for calculations of pharmacokinetic parameters [22].

For the IAM-HPLC analysis IAM.PC.DD 2 column was used. Mobile phase was phosphate buffer modified with different volumes of methanol. For the HSA-HPLC analysis Chiral HSA column and gradient of phosphate and 2-propanol were used. IAM-HPLC analysis showed that ionization of polyphenolic under physiological pH was influencing the retention of the substance due to ion-ion interaction. The strongest interactions were in the group of flavonoids (kaempferide log *k*_wIAM _= 3.581) and lower for phenolic acids (log *k*_wIAM _= 1.35–1.68).

**Figure 11:**
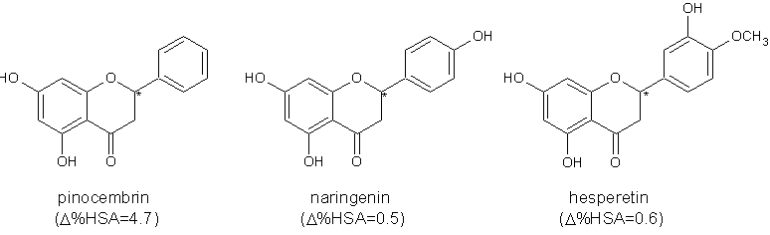
**Structure and differences in HSA binding of enantiomers**.

From the results of HSA-HPLC analysis it has to be noticed that all the flavonoids have percentage of bonded fraction grater than 95%, which can have repercussions to the interactions with drugs. Binding to HSA was higher for myricetin (%HSA = 99.8) from the group of flavonoids and the lowest for caffeic acid (%HSA = 81.8) from the phenolic acids analyzed, both having the greatest number of hydroxyl groups. Enantiomers could have been differentiated using this method, as expected. This is the case with flavanons shown on Figure 11.

**Figure 12:**
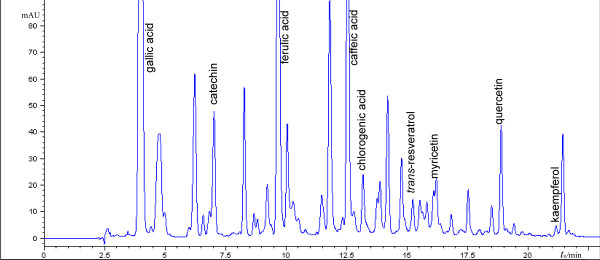
**HPLC chromatogram of wine sample Postup registered at 280 nm**.

HPLC analysis of wine was conducted on Agilent 1100 using Zorbax StableBond C18 column [5]. Binary mobile phase of eluents A (phosphoric acid (c = 0.02 M, pH = 3.0 adjusted with triethylamin) : methanol = 90 : 10 *v/v*) and B (phosphoric acid (c = 0.02 M, pH = 3.0 adjusted with triethylamin) : methanol = 10 : 90 *v/v*) was used with the gradient stated in Table 8. Flow rate was 1 mL/min at 35°C. 15 standards of polyphenols were used; their identification was based on retention time and UV spectrum, and quantification was done at 280 nm. HPLC analysis of samples of wine has shown discrepancy in results compared to TLC. This has been described in literature [25,26] and can be explained by lower limit of detection and possibility of overleaping the peaks in HPLC. To illustrate this comparative results for Pelješac sample are shown in Table 9. Chromatogram of wine samples from Croatia obtained by HPLC analysis is shown on Figure 12.

**Table 8:** Gradient of mobile phases used for HPLC analysis of wine.

time (min)			
			
mobile phase	0	25	30
%A	85	15	5
%B	15	85	95

Legend: *only caffeic acid degraded up to 9.4% in 120 minutes, but as the analysis lasts less, this was not of great significance; CA: caffeic acid; IFA: isoferulic acid; PME: pinocembrin-7-methylether; RSD: relative standard deviation.

**Table 9:** Comparison of the results obtained by HPLC and TLC analysis for Pelješac sample.

	**gentisic acid**	**gallic acid**	**cinnamic acid**	***o*-coumaric acid**	***m*-coumaric acid**	***p*-coumaric acid**	**caffeic acid**	**ferulic acid**	**chloragenic acid**	**catehin**	**quercetin**	**kaempferol**	**myricetin**	**quercitrin**	**apigenin**	**naringenin**	***trans*-resveratrol**
**HPLC**	na	8.4	na	-	-	-	5.0	0.7	-	4.1	4.1	0.4	-	-	-	-	0.8
**TLC**	48.23	8.92	36.56	na	-	-	3.50	0.93	na	na	5.06	-	na	na	-	-	na

Results expressed as mean of mass concentration in mg/L (na – not analyzed, - – not identified).

### Spectrophotometry

The Folin-Ciocalteu method was used for quantification of total polyphenols according to the method described by Slinkard and Singleton [27]. The method is based on the reduction of MoO^4+ ^to MoO^3+ ^that is detected by color change from yellow to blue; measured at 765 nm. The results were expressed as equivalents of gallic acid from the calibration curve.

**Figure 13:**
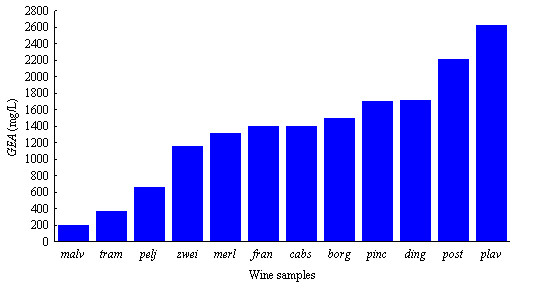
**Total polyphenols in wine samples as equivalents of gallic acid (GAE, mg/L) **[5]. Codes for wine samples: *malv *– Malvazija; *tram *– Traminac; *pelj *– Pelješac; *zwei *– Zweigelt; *merl *– Merlot; *fran *– Frankovka; *cabs *– Cabernet Sauvignon; *borg *– Borgonja; *pinc *– Pinot crni; *ding *– Dingač; *post *– Postup; *plav *– Plavac.

The results for determination of total polyphenols, according to the Folin-Ciocalteu method have shown that white wines (malvazija (*malv*), Traminac (*tram*), Pelješac (*pelj*)) have a lower content of polyphenols [5]. Red wine contains more polyphenols than white wine. This may be ascribing to the fact that making of white wine requires the removal of the skins after the grapes are crushed. Wines containing higher contents of polyphenols are those from the south of Croatia (Plavac(*plav*), Postup (*post*), Dingač (*ding*)) (Figure 13). These findings have been confirmed by TLC analysis.

Free stable radicals DPPH^· ^and ABTS^·+ ^were used to assess the antioxidant activity. The method is based on lost of violet/blue color of radicals in presence of substance capable to accept free electron. Discoloration was measured on spectrophotometer at 518 and 730 nm respectively [22]. The spectrophotomety assays, using stable free radicals to assess the antioxidant activities have proven reliable and precise. As most of the suggested procedures could not differentiate similar samples, kinetics of different concentrations of propolis samples has been studied and antioxidant efficiency was introduced (AOE). DPPH^· ^radical was more stable than ABTS^·+ ^and results of the DPPH^· ^assay showed better reproducibility. Based on the content of flavonoids and phenolic acids identified and quantified by HPLC analysis we obtained the following correlation:

(12)

### Quantitative Structure-Activity Relationship

Experimental data used for QSAR analysis was taken from the literature. Wiener index, connectivity index, Balaban index, Balaban-type indices from atomic number, mass, van der Waals, electronegativity and polarizability weighted distance matrix, information-theoretic index, Shultz index, together with molecular weight, n-octanol/water partition coefficient, van der Waals volume, molar refractivity and polar surface area of polyphenols were calculated using TAM [28], HyperChem 8.0 Evaluation software, PCLIENT, Dragon 3.0. Four groups of 3D descriptors were used: geometrical, GETAWAY (Geometry, Topology and Atom Weights Assembly), 3D-MoRSE and RDF (Radial Distribution Function). Linear, polynomial and multiple linear regression analysis was conducted using Statistica 6.0 [16].

**Figure 14:**
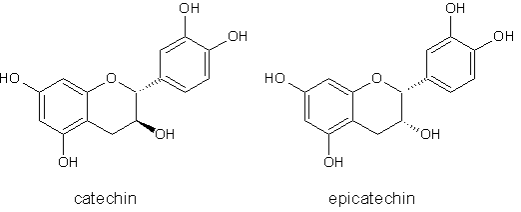
**Structures of catechin and epicatechin**.

Statistically significant QSAR models for lipid peroxidation inhibiting effects of flavonoids were obtained by polynomial and multiple regression using lipophilicity, Balaban index, Balaban-type index and 3D GETAWAY descriptor. The reason for selecting the 3D descriptors was the possibility of discriminating stereoisomers, like catechin and epicatechin (Figure 14).

The best model that includes 3D descriptor *H*_7_(p) in combination with Balaban-type index from mass-weighted distance matrix (*J*_m_) was chosen by the best-subset regression:

(13)

*H*_7_(p) is an autocorrelation descriptor calculated for 3D-spatial molecular geometry based on lag (topological distance) and weighted by atomic polarizabilities. This descriptor belongs to the group of H-GETAWAY descriptors that have been calculated from the molecular influence matrix H. These descriptors are sensitive to significant conformational changes and to the bond lengths that account for atom types and bond multiplicity.

This QSAR investigation of wine polyphenols could be extended to a larger number of substances, including anthocyanins and dimeric procyanidins as the most common polyphenols in wine.

### Clinical Trial [29]

An *in vivo *study has been conducted on 47 healthy women and men in order to investigate whether daily intake of powdered propolis extract during 30 days has any influence on antioxidative status based on the following blood parameters: activity of superoxide dismutase, glutathione peroxidase and catalase, concentration of plasma malondialdehyde, total cholesterol, low- and high-density lipoprotein cholesterol, triglycerides, glucose, uric acid, ferritin and transferrin, together with routine red blood cell parameters.

**Figure 15:**
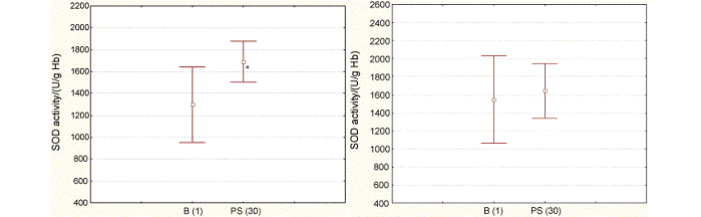
**Antioxidant activity of propolis *in vivo***. Activity of superoxide-dismutase (SOD) (U/g Hb) during the study: a) in the men test group and b) in the women test group. Data are expressed as means ± S.D.; B (1) represents the baseline on the 15^th ^day prior to the study and PS (30) represents propolis supplementation on the 30^th ^day. The symbol (*) denotes statistically significant change [29].

This was the first reporting of the effects of prolonged propolis supplementation on redox-status of human organism. The benefit of propolis use was only shown in male population demonstrating reduction in free-radical-induced lipid peroxidation as well as increase in activity of superoxide dismutase. A 23.2% decrease in malonaldehyde (degradation product of peroxidation of polyunsaturated fatty acids) concentration and a 20.9% increase in superoxide dismutase activity (first and most important line of antioxidant enzyme defense) were observed (Figure 15).

Absence of the same results in women was probably due to uncoordinated menstrual cycles. Hence, estrogens are powerful antioxidants, modulators of antioxidant enzyme expression and levels of lipid peroxidase and lipoproteins.

## Summary and conclusion

In the last decade interest on nutraceuticals and natural medicinal products is constantly growing. The market is full of antioxidant formulations of wide variety of sources. For the registration of nutraceutical as natural medicinal product analytical procedures have to be developed, product has to be standardized and their functionality and beneficial effects have to be demonstrated. The goal of our studies was standardization of Croatian propolis extracts as a rich sample of flavonoids and phenolic – active components to which pharmacological (antioxidant) activity is attributed. Parallel research on wine samples was done, as wine represents another sample rich in polyphenolics that has been used as constituents e.g. iron wine, tonics.

### Identification and quantification of flavonoids and phenolic acids

Although the methods for identification and quantification of propolis samples using HPLC combined with mass spectrometry (MS) and nuclear magnetic resonance (NMR) have been developed and alternative was provided in capillary electrophoresis coupled with MS, these are rather expensive, not readily available and inappropriate for routine analysis [30,31]. Thus we have decided for TLC method combined with HPLC-diode array detection (DAD) method. Out of 11 mobile phases available from literature based on numerical taxonomy chloroform : methanol : formic acid in volume ratio 44 : 3.5 : 2.5 was selected as the most appropriate for TLC identification of 9 polyphenols [17]. Better separation for identification was achieved using 2D-TLC chromatography, although for quantification we decided for high performance TLC plates using which clear identification and quantification of 7 polyphenols (namely: *p*-coumaric acid, caffeic acid, chrysin, tectochrysin, pinocembrine-7-methyl ether, isoferulic acid) was performed [18,19]. Major limiting factor of TLC is length of the plate, so for better separation HPLC was used.

Total content of polyphenols was determined using Folin-Ciocalteu method. As we have done identification using liquid chromatography we did not separately analyze total content of phenolic acids and flavonoids which can be done using well known procedures described in Ph. Eur. and Christ and Müller, respectively [32,33].

### Pharmacokinetic (ADME) parameters of flavonoids and phenolic acids

Passive transport through membranes is mainly determined by the lipophilicity of the substance. The most commonly used experimental value of lipophilicity is chromatographic parameter *R*_M_. This parameter was determined by TLC as well by HPLC and to assess lipophilicity within the group of polyphenolics. As this parameter cannot distinguish isomers, additional parameter *Φ*_0 _was used differentiating sakuranetin and isosakuranetin.

Using ChemSilico software plasma protein binding of flavonoids was found rather high (85–96%) compared to phenolic acids (70–86%). High values of flavonoid binding are in accordance to the experimental date e.g. for quercetin (99%) [34]. Passage through blood-brain barrier was negative which is expected for compounds having acidic phenolic and carboxylic groups. Absorption was mainly greater than 60%, but this has to be taken with dose of doubt, as experimental data is contradictory [35].

Chemical interaction with most commonly used non-steroidal anti-inflammatory drugs (NSAID) and vitamins where characterized on TLC plate. The interactions were most common with acetylsalicylic acid. At the moment this has no practical value as further *in vivo *studies have to demonstrate applicability of this model [21].

Interactions of polyphenolics with artificial membranes were probably based on ion-ion and passive diffusion as interaction showed greater partition coefficient for flavonoids than phenolic acids. Binding on HSA-column was higher compared to ChemSilico predictions and more in accordance to experimental data obtained *in vivo*.

### Antioxidant activity of flavonoids and phenolic acids

Antioxidant activity of polyphenolics is well known. To assay the antioxidant activity different methods are used: electron paramagnetic resonance (EPR) spectroscopy, chemiluminescence, enzyme assays... Most often used and readily available is spectrophotometric method using stable free radicals. DPPH^· ^and ABTS^·+ ^were used for spectrophotometry as well TLC *in situ *for analysis of total antioxidant capacity of propolis. The most active propolis comes from coastal Croatia [22].

QSAR analysis of polyphenolics based on Trolox test date from literature pointed out that antioxidant activity of polyphenols as hydrogen donating free radical scavengers, is closely related to their chemical structure, especially with the number and arrangement of free hydroxyl groups of polyphenol skeleton [16].

*In vivo *study of propolis prolonged used showed beneficial in male population demonstrating reduction in free-radical-induced lipid peroxidation as well as increase in activity of superoxide dismutase. Production of malonaldehyde (degradation product of peroxidation of polyunsaturated fatty acids) reduced and activity of superoxide dismutase (first and most important line of antioxidant enzyme defense) was increased [29].

Antioxidant supplements are flooding the market. Asking pharmacist for a new product – extract of the plant coming from exotic country, will usually end with the answer "its antioxidant and thus good for your health". The necessity of standardization natural antioxidant products made us write this minireview-providing basis for standardization of natural antioxidant products rich in polyphenols using simple and readily available techniques based on our research on propolis and wine.

## Abbreviations

ABTS: 2,2'-azino-bis(3-ethylbenzothiazoline-6-sulphonic acid; ADME: absorption, distribution, metabolism, excretion; AOE: antioxidant efficacy; AU: arbitrary unit; BBB: blood brain barrier; 3D descriptors: three-dimensional descriptors; 2D-TLC: two-dimensional thin layer chromatography; DAD: diode array detection; *DP*: discriminating power; DPPH: 2,2-diphenyl-1-picryl-hydrazyl; EPR: electron paramagnetic resonance; GAE: gallic acid equivalent; GETAWAY: geometry, topology and atom weights assembly; GIT: gastrointestinal tract; HSA: human serum albumin; HPTLC: high performance thin layer chromatography; IAM: immobilized artificial membrane; MS: mass spectrometry; NMR: nuclear magnetic resonance; NSAID: non-steroidal anti-inflammatory drugs, OTC: over-the-counter; RDF: radial distribution function; *R*_F_: retention factor; *R*_M_: chromatographic parameter of lipophilicity; RP-HPLC: reverse phase high performance liquid chromatography; RP-TLC: reverse phase thin layer chromatography; SOD: superoxide-dismutase; UV: ultraviolet.

## Competing interests

The authors declare that they have no competing interests.

## Authors' contributions

All authors contributed equally to this work, read and approved the final manuscript.
